# Towards better Diabetes Data Rights: Data protection, data ‘ownership’, and developing a patient charter

**DOI:** 10.1111/dme.70160

**Published:** 2025-11-06

**Authors:** Ruby Reed‐Berendt, Jessica Bell, Louise Hatherall, Holly Hayes, Shane O'Donnell, Muireann Quigley

**Affiliations:** ^1^ Birmingham Law School University of Birmingham Birmingham UK; ^2^ Edinburgh Law School University of Edinburgh Edinburgh UK; ^3^ School of law University of Warwick Coventry UK; ^4^ College of Business University College Dublin Dublin Ireland

**Keywords:** clinical diabetes, data ownership, data protection, diabetes data

## Abstract

An important implication of the increasing availability and use of technologies to support care for people with diabetes is that huge quantities of data are now being generated about them on a routine basis. This has led to calls for people with diabetes to have greater access to and control over how ‘their’ health data are used. In this article, we analyse some of the legal challenges arising in this context. Focusing on the United Kingdom and European Union, we point to the lack of a clear legal framework to balance the different interests in the context of diabetes data, noting that calls for ‘data ownership’, while understandable, do not fit easily within existing legal frameworks and would not offer the kind of protections that some within the diabetes community are advocating for. We offer suggestions for supporting such calls. We first note the existing, but perhaps under‐utilised, legal mechanisms available to people with diabetes over their data, such as data subject rights under the General Data Protection Regulation. Then, drawing inspiration from existing and emergent frameworks, we argue that the interests of people with diabetes could be bolstered by something like the Diabetes Data Rights Charter currently being co‐developed by researchers and members of the diabetes community.

**What's new?**

- Drawing on research with members of the diabetes community, this article examines calls for people with diabetes to have greater access to, and control over, their diabetes data.
- Data protection legislation in the United Kingdom and the European Union are examined, along with the concept of ‘data ownership’, with a view to demonstrating how critical limitations within the law mean that, in practice, people's interests in their diabetes data may not be adequately protected.
- The article underscores the need for other means to address the challenges around access to and control of diabetes data, while also ensuring proper privacy and security provisions are in place.
- It highlights the novel Diabetes Data Rights Charter currently being co‐developed by researchers and members of the diabetes community, outlining how the Charter is useful for both people with diabetes and other key stakeholders.

## 1 INTRODUCTION

Data generated by diabetes devices, such as continuous glucose monitors (CGMs), insulin pumps, smart pens and hybrid closed loop systems are fundamental to diabetes care. Despite this, people with diabetes (PwD) (and those that care for them) often lack full access to, and control over, real‐time data affecting them. This issue has become a major concern for device users,^1^ reflected in movements such as the #WeAreNotWaiting movement, a self‐organised community of PwD who have been creating safe and effective open‐source tools to better manage their condition for over 10 years. These include tools such as Nightscout and open‐source automated insulin delivery (OS AID) systems. Nightscout is ‘is an open‐source cloud application used by PwD and parents of children with diabetes to visualize, store, and share the data from their CGM sensors in real‐time’.^2^ OS AID systems are comprised of OS software which connects a person's CGM and insulin pump to enable near real‐time (semi‐)automated insulin delivery. The OS component of this has been, and continues to be, developed, maintained and supported by PwD for PwD. These innovations arose partly from frustration with device companies' restrictive policies that limit user access to, and control over, their (device) data.^3^

Concerns around data access and control are not just confined to open‐source solutions. The increasing availability—and use—of both commercially available and open‐source diabetes technologies means that huge quantities of data are now being generated on a daily basis. For example, CGMs generate glucose values in near real time every 1–5 min, thus creating a huge number of data points.^4^ Concerns around the generation, collection and use of diabetes‐related data, both within and outwith the clinical context, are garnering more attention. Notably, claims that PwD ought to have more control over and access to ‘their’ data are sometimes expressed in terms of ownership (echoing similar calls in other health contexts).^5^ For example, in research we conducted with members of the diabetes community, one participant noted that ‘the person with the wire in their arm deserves to know what that data is and own that data’. Meanwhile, another said ‘any information of a device I'm wearing, whether it's coming from my physiology or into it, needs to be ours’.

In this article, we analyse challenges and opportunities regarding diabetes technologies and data. In Section one, we outline some of the key issues that exist in relation to (the use of) diabetes data. In Section two, we outline how the law governs data and note that existing legal mechanisms under data protection legislation—namely data subject rights under both the United Kingdom's (UK) and European Union's (EU) General Data Protection Regulation (GDPR)—have the potential to support PwD with regards to their data. However, we also note that limitations in scope and enforcement can mean that, in practice, these frameworks may not be enough to protect the interests of PwD. We also discuss how calls for ‘data ownership’ do not fit within existing legal frameworks for data governance. Given this, in Section three, we argue that PwD interests could be bolstered by something like the Diabetes Data Rights Charter we are co‐developing with the diabetes community. This Charter will articulate a set of principles for industry, healthcare institutions, and other stakeholders to consider when developing diabetes technologies so that PwD can, safely and securely, access and exercise control over their diabetes data in a manner that better meets their needs. It is intended as a practical tool to guide responsible, user‐centred development of diabetes technologies, ensuring that innovation delivers benefits equitably to those most affected. These principles also aim to support innovation by improving data sharing (and integration) pathways across devices and platforms, expanding market reach and fostering innovation partnerships (including co‐development with PwD). Importantly, it responds directly to concerns raised by PwD and parents of children with diabetes about the lack of meaningful access to and control over their data.

## 2 DIABETES DEVICES AND DATA: SOME CHALLENGES

Collectively, diabetes devices generate and store a wide range of data including glucose values, mealtime and correction boluses, carbohydrate intake calculated and inputted by the user, basal rates, temporary targets in response to physical activity, hormonal change, hypoglycaemic events and much more. These data are useful and important in numerous contexts, such as device development, manufacturing, research and, of course, day‐to‐day diabetes management. They are also useful to a wide range of potential stakeholders, including PwD, their families and carers, healthcare professionals, researchers and manufacturers. However, diabetes data is often ‘locked‐in’ to specific manufacturers or specific programmes, limiting user choice to those which a manufacturer makes for them. This includes limits on what data can be downloaded and moved to PwD’s preferred providers or platforms. As such, users have little to no control over the data generated by their own bodies. Device vendors, for their part, have argued this closed approach to medical device innovation is necessary to meet cybersecurity requirements set by regulatory bodies. As such, there are pressing questions about who ought to get access to/control device‐generated data and in what form, as well as who should determine this. Additionally, there are questions about how data security and privacy can be adequately protected and balanced against considerations of access and control.

Answering these is not a simple matter. Some members of the diabetes comunity feel that their needs and interests are currently not being appropriately considered, and that existing frameworks place too much control in the hands of industry.^6^ In particular, PwD are concerned that access to data may be hampered by manufacturer policies around cybersecurity and data governance, as well as regulatory frameworks which can (in practice if not in principle) work to restrict such access.^7^ As various commentators have recognised, integration and communication between various devices and systems is required in order to properly harness and realise the potential of diabetes data and for PwDs to manage their condition.^6^, ^8^ The ability to move data, in a (re)usable form, between different data/device management platforms is vital to such integration.

Given this, there are increasing calls for ‘openness’ in the diabetes ecosystem, with a focus on transparency, accessibility and control regarding the use of health data.^7^ As noted earlier, this is sometimes framed in terms of data ownership. While such calls are understandable, as we will see in the next section, the law does not recognise ownership of data per se, and so approaching the issues from this standpoint may not offer PwD the kinds of rights over their data, which they are after. Despite the desire for more openness in the diabetes ecosystem, it bears emphasising that diabetes data remain highly personal and sensitive. Thus, ensuring that data are appropriately protected against misuse and that data privacy can be guaranteed is of the utmost importance.^9^ Significantly, groups which continue to be disadvantaged in their access to diabetes technology and life‐saving care at both global and local levels may be more vulnerable to harms arising from data misuse, and therefore more likely to have concerns about inappropriate data use and sharing.^8^, ^10^, ^11^

## 3 DATA PROTECTION AND ‘DATA OWNERSHIP’ IN THE UNITED KINGDOM AND EUROPEAN UNION

Data protection legislation is the principal way in which the law governs and regulates the use of personal data, including health data. In the United Kingdom, this is the UK General Data Protection Regulation (GDPR), part of retained law post‐Brexit which, for the time being at least, remains reflective of the provisions set out in its counterpart: the EU GDPR. The purpose of the GDPR is to protect the fundamental rights and freedoms of individuals, particularly in relation to personal data. It sets out both the key principles (e.g., transparency and fairness) and necessary conditions for the processing of personal data, including health data, which is classified under both the UK and EU GDPR as ‘special category’ data^12^ due to its sensitivity.

Special category data may only be processed under strict conditions—most commonly with explicit consent, but also under limited exceptions such as processing for scientific research or for public health purposes where there is an overriding public interest. These alternatives, while potentially more robust in some contexts, can be unevenly applied and poorly understood, potentially contributing to confusing and inconsistent governance.^13^ Where consent is used, it must be explicit and based on a thorough understanding of the specific purposes for which the data are to be used. While not providing PwD with access per se, this is one mechanism, through which they can exercise control over their health data, including the right to withdraw consent if they find the uses unfavourable. However, the requirement for explicit consent often results in lengthy documentation, which not everyone is motivated or has the wherewithal to read before agreeing to access diabetes management technologies.

Under the GDPR, data subjects also have a suite of rights that, in principle at least, enable them to do certain things with their data once it has been collected by someone else. These were introduced to address the imbalance of power that exists between those collecting, storing and using data (data controllers/processors) and those providing that data (data subjects). Key among these for our purposes is the ‘right of access’ and the ‘right to data portability’.^14^ Access rights under the GDPR mean that, in principle, data subjects ought to be provided with a copy of any data, including health data that are being used by the person holding the data. Furthermore, it is incumbent on the data controllers/processors to provide clear information about how to access data at the time at which it was collected. Relatedly, the right to portability consists, again in principle, of a right to receive personal data in a structured, commonly used, machine‐readable and interoperable format. This allows individuals to reuse their personal data for their own purposes and to transfer data without impacting on usability (although this only applies to certain processing purposes).^15^

GDPR requirements may seem promising, but limitations remain. The right to data portability, for example, does not require data controllers to maintain compatible systems and has been criticised for failing to guarantee coverage of all required data, including continuous, real‐time access.^16^ So, in practice, PwD may not obtain all relevant data or be able to access it in the format they need. Moreover, enforcement of data rights is not always straightforward. While the UK and EU GDPR both place accountability obligations on data controllers, individuals are often left without adequate institutional support to navigate complex data governance systems or enforce their rights effectively. Indeed, issues relating to understanding what data are held and where (necessary to pursue enforcement of particular rights) have been identified in different data contexts^17^ and were raised as a concern by PwD during our research. Additionally, there can be uncertainty over which legal jurisdiction covers the data processing. As a result, the predominant burden of enforcement is placed upon individuals.^18^ All of this can make the enforceability of the rights contained within the GDPR at best impractical and, at worst, impossible. While this issue affects people in a wide variety of data contexts, the impacts are felt more sharply by those, such as PwD, where access to data has direct, ongoing and everyday implications for their health.

Given some of the limitations of data protection law, appeals ‘data ownership’ may have a certain ‘intuitive appeal’.^19^ If someone says to us that they own something or it is their property, at a general level we understand the types of claims they are making. Legally, if they own a thing, they have a range of powers to use and control (the use of) it. This may include, for instance, powers to determine the use of the thing, to exclude others from using the thing, and to transfer it (or a part of it) to another. Importantly, for the law, property rights are (in principle at least) enforceable against all others.^20^ It is therefore easy to see why there have been calls for ‘data ownership’ by PwD, as well as across other health contexts such as genomics^21^ and brain cancer.^22^ However, while property law provides a good framework for dealing with tangible items, it is less well suited for dealing with intangibles such as data.

First and foremost, as noted in a recent report on data ownership, although this term is ‘often used as a convenient legal shorthand to describe that a certain party has a certain control over specific data’, this term is not used in legislation.^23^ Whether a legal concept of data ownership should be developed and incorporated into legal instruments is the subject of ongoing debate.^24^ But to date, the general consensus is that we do not own our health data. This might come as a surprise, especially when considering that we also do not own other types of health data that we might consider to be most intrinsically ‘ours’, such as our DNA and genetic data.^21^

Second, while the information generated by devices will contain personal health information about the patient/device user, it is not created solely by them but in conjunction with a device. This can give rise—rightly or wrongly—to claims of ownership or rights by manufacturers who might argue they have ‘generated’ that data,^19^, ^20^ or that sharing said data might reveal proprietary information (such as trade secrets). These competing rights or interests make it more challenging for patients to claim (sole) ownership of the data.

Third, data—unlike tangible items—can be accessed, used and reused by multiple parties in a non‐mutually exclusive manner. Although someone else cannot use a pen at the same time I am using it (regardless of who owns it), the same is not true of data. PwD, researchers, manufacturers and so on could all, at least in theory, have access to the same data(sets).^23^ An ownership paradigm, which would involve granting *exclusive* rights to one or multiple parties, may therefore cause needless harm by diminishing the legitimate opportunities for others to use it.^25^

The problem is that, although legally‐speaking, neither manufacturers nor users “own” the data in a property sense, manufacturers typically exercise de facto control through technical design and contractual means, as well as often holding trade secret protections and intellectual property rights in databases. This creates a power asymmetry that limits access for users, researchers, and others. Given all of this, what should be done? In the final section, we consider how a Diabetes Data Rights Charter, currently being co‐developed with people with diabetes, might respond to these challenges and complement emerging regulatory initiatives, while centring the voices of those most affected and strengthening their ability to advocate for meaningful control over data.

## 4 EMERGING REGULATORY TRENDS AND DEVELOPING A DIABETES DATA RIGHTS CHARTER

The problems around encouraging openness and access to diabetes data while also taking account of privacy and security are widely recognised in different data contexts; for example, in debates regarding ‘Big Data’^11^ and genomic data.^21^ These debates place particular emphasis on ongoing power imbalances between those who control and benefit from the data and those to whom the data relates. A key challenge, as noted earlier, is that—despite the rights contained in the GDPR—data subjects often lack mechanisms to effectively control their data. Moreover, the lack of control can mean that the use of, and knowledge generated from, datasets does not reflect the priorities of particular condition communities and may not be used in ways that are valuable and useful to those communities. All of this speaks directly to the concerns of PwD when it comes to their data. Being required to work within paradigms around access and security set by device manufacturers and other more powerful stakeholders means that PwD may not gain the benefits *they* desire from using diabetes technologies.

Recent regulatory developments in the EU—including the EU Data Act^26^ and the European Health Data Space (EHDS)^27^—are starting to reshape health data governance in ways that may partially address some of the challenges outlined above. The EU Data Act introduces obligations for manufacturers of connected medical devices (including CGMs and insulin pumps) to ensure users can access and share data generated by those devices. The EHDS, meanwhile, seeks to harmonise access to electronic health data across EU Member States, supporting both patient rights and secondary uses such as research.^28^ These initiatives represent progress, yet remain limited. They do not directly apply to non‐EU Member States (including the UK), focus primarily on technical data sharing and secondary use rather than meaningful, user‐centred control and leave significant gaps in enforcement and practical implementation.^29^

In light of these concerns, and arising out of discussions with PwD, we set out to co‐produce a Diabetes Data Rights Charter with members of the diabetes community to help address the challenges identified, and importantly, centre the voices and interests of those most affected. Drawing inspiration from other user‐centred charters and existing ethics frameworks, the Charter will articulate a set of principles that key stakeholders should consider when developing diabetes technologies so that PwD can, access and exercise control over their diabetes data in a manner that better meets their needs. The Charter is an opportunity to develop principles to guide diabetes data governance moving forward in a way that aligns with the practical realities of those who use diabetes technologies. Thus far, principal points—which were generated through a series of roundtables attended by PwD and parents of children with diabetes—have centred on data access, control, benefit and the need for industry transparency and accountability.

Regarding data access, participants emphasised the importance of access to their diabetes data safely and securely, in real time and in a format they can use. Access, in this context, includes data generated by devices, as well as data held by healthcare professionals and institutions. Key to these discussions was a need for diabetes data to be complete (e.g. include relevant meta‐data), available in ways that were useful for the individual (e.g. in their own language) and genuinely portable, that is, exportable in a manner that is useful to them personally. This could include, for example, being in a format that is readable for Bluetooth devices, or capable of being sent to a platform of their choosing.

In relation to control, participants at the roundtables emphasised the importance of having meaningful consent and control over the use of diabetes data. Vitally, the contours of this consent should enable PwD to choose what data is shared and with whom. Participants stressed that this should not be ‘all or nothing’: PwD should be entitled to choose to share their data with some individuals or organisations and not others (or not at all) without losing access to their devices and associated features. Participants ideally want consent to be ‘opt‐in’ rather than ‘opt‐out’, and for industry stakeholders to develop clear pathways to establish ongoing consent to the use of their data.

Threaded throughout the roundtable discussions was also the importance to PwD of responsibility, accountability, transparency and trust. Ensuring that any diabetes data held were useful and accurate was emphasised, with PwD arguing for an associated right to rectify data that are inaccurate. The discussion moreover underscored many of the criticisms laid out above, including a lack of transparency on what data are held, how they are used and the ways in which models (including new machine learning models) are trained and developed. This obstructed view of how data are stored, utilised and developed acts as a barrier to trust for PwD. Participants want greater oversight of the myriad uses of their diabetes data, including the right to know when such data are destroyed.

Based on the roundtables, and in conjunction with a working group of PwD, a set of principles for the Charter will be drafted and refined. It is anticipated that the Charter will increase awareness around the existing mechanisms available for PwD to access and control their data. It could also be used as an advocacy tool to empower PwD when engaging with stakeholders—such as device manufacturers—and organisational officials responsible for data governance matters—such as Data Protection Officers and the Information Commissioner's Office—should PwD wish to make a complaint about matters concerning their data.

The Charter could also benefit other stakeholders within the diabetes ecosystem, such as device vendors. By aligning with Charter principles, industry stakeholders can reduce reputational and legal risks, differentiate themselves in a competitive market and foster trust among increasingly data‐aware consumers. Improved interoperability and data portability can enable smoother integration across platforms, expanding market reach and fostering innovation partnerships. Co‐producing technologies with users can streamline development and increase uptake, while a shared governance framework can reduce fragmentation and support a more stable innovation environment. By promoting legal and ethical compliance, the Charter can encourage companies to go above and beyond the minimum standards required by law. Adherence to higher standards can foster greater transparency and trust for the benefit of both industry and other stakeholders, particularly PwD. Engaging with patient‐centred principles from the outset can streamline development by improving user satisfaction, reducing friction, and preempting regulatory or ethical challenges.

## 5 CONCLUSION

The potential benefits of diabetes data for improving the health and wellbeing of PwD are manifold. However, the challenges to realising these are similarly varied, particularly given the competing interests of different stakeholders. Key sticking points centre around questions of who has, or indeed ought to have, access to data, who ought to determine how it can be used, and issues of data privacy and security. We saw that current legal frameworks around data protection have in‐built limitations and are, therefore, inadequate to the task of properly upholding PwD interests when it comes to their data. Moreover, appeals to data ownership, while on the face of them attractive, have very limited legal bite.

A Diabetes Data Rights Charter—by articulating principles grounded in the lived experiences of PwD—can complement existing legal frameworks to help address gaps in practice, and provide a global reference point for industry, healthcare systems and policymakers. In doing so, this would support both innovation and practical implementation in diabetes care. Far from delaying innovation, the Charter aims to foster trust and transparency, which are essential for the successful uptake and long‐term sustainability of new diabetes technologies. By having a Charter focused specifically on diabetes data, communities will be in a stronger position to assert and defend their interests, including calls for greater access to and control over data relating to their health.

## FUNDING INFORMATION

Research relating to this paper has been supported by the Wellcome Trust (grant no. 212507/Z/18/Z, PI: MQ), the European Union (101064383) and a Research England quality‐related research grant. The development of the Charter is co‐funded by a University of Birmingham Institutional Impact Fund and an Arts and Humanities Council Impact Acceleration Award [grant no. AH/X003388/1].

## CONFLICT OF INTEREST STATEMENT

The authors declare no conflicts of interest.

## ETHICS STATEMENT

Approval for research relating to the co‐development of the Diabetes Data Rights Charter has been obtained from the University of Birmingham's Humanities and Social Sciences Ethics Committee (ERN_3095‐Aug2024).
